# A Service Evaluation of Admission Outcomes Across One Year in a Specialist Dementia Intensive Care Unit

**DOI:** 10.1192/bjo.2026.11625

**Published:** 2026-06-30

**Authors:** Ameedat Oguntunde, Murphy Ositaufere, Mathuri Tharmapoopathy, Josef Mahdi

**Affiliations:** Sussex Partnership NHS Foundation Trust, Worthing, United Kingdom

## Abstract

**Aims:**

Specialist dementia inpatient units provide care for individuals with complex neuropsychiatric and behavioural needs. Understanding admission outcomes within these settings is essential for service planning and optimising patient flow. The aim of this service evaluation was to evaluate admission outcomes over a one-year period for all inpatients admitted to a 30-bed specialist dementia intensive care unit including diagnoses, length of stay, discharge destinations and mortality.

**Methods:**

A retrospective service evaluation was conducted of all inpatients admitted to Sage, Rosemary and Thyme ward in the Forget Me Not Unit in Worthing between March 2024 and March 2025. Data were extracted from electronic patient records and anonymised prior to analysis, which included demographic data, diagnoses and dementia subtype, legal status upon admission and discharge, proportion of prescribed psychotropic medications, length of stay, time taken to be declared medically optimised for discharge (MOFD), discharge destination and mortality. Descriptive statistical analysis was performed for each ward and for the unit overall.

**Results:**

There were 88 inpatient admissions across all three wards within one year (34% female). This inpatient cohort had a mean age of 80 years (median 81 years) with most admissions being facilitated under section 2 of the mental health act (76%) and a minority under section 3 (15%). Alzheimer’s disease was the most common diagnosis at discharge (42%) followed by mixed dementia (28%) and a smaller proportion of vascular dementia (7%), Lewy body dementia (6%) and frontotemporal dementia (2%). On average, patients were declared MOFD after 63 days (median 49 days) and were subsequently discharged after a mean of 61 days (median 59 days) leading to an average length of stay of 124 days (median 113 days). Approximately 55% of all patients were discharged to a care home, 15% were discharged home and 5% died during their admission. Mortality within twelve months post-discharge was high at 22% with half of these deaths occurring within three months of discharge.

**Conclusion:**

This service evaluation highlights the complexity of care, prolonged admissions and high post-discharge mortality associated with specialist dementia inpatient services. Delays between patients being declared medically optimised and subsequently being discharged from the unit highlight an important area for service improvement, particularly within adult social care. These findings offer a useful baseline for future comparisons which may guide local quality improvement, workforce planning and multidisciplinary team working.

